# Parishin B Attenuates PTZ-Induced Seizures in Zebrafish and Is Associated with Neurotransmitter Balance and ACLY-Related Metabolic Pathways

**DOI:** 10.3390/metabo16040275

**Published:** 2026-04-18

**Authors:** Meng Sun, Haida Liu, Zhiying Hou, Qiong Wang, Wu Zhong

**Affiliations:** 1 Affiliated Medical College Emergency Department, Southwest Medical, Luzhou 646000, China; 20230299120149@stu.swmu.edu.cn (M.S.); 20250299120171@stu.swmu.edu.cn (H.L.); 2 Institute of Food Science and Technology, Chinese Academy of Agricultural Sciences, Beijing 100193, China

**Keywords:** Parishin B, epilepsy, zebrafish, integrated proteomics and metabolomics, ATP-citrate lyase (ACLY), ferroptosis, neuroinflammation

## Abstract

**Highlights:**

**What are the main findings?**

- Parishin B is associated with attenuation of PTZ-induced seizures in zebrafish larvae through multi-target mechanisms, including alterations in GABAergic and monoaminergic neurotransmitter balance, suppression of neuroinflammation, and reduction of oxidative stress.
- Integrated metabolomic and proteomic analyses reveal that Parishin B correlates with alterations in energy lipid metabolism involving the ACLY-mediated axis, potential modulation of ferroptosis-related pathways, and normalization of disrupted nucleotide and ribosomal pathways.

**What are the implications of the main findings?**

- Parishin B represents a promising multi-target candidate for anti-epileptic therapy, with potential relevance to both acute seizure symptoms and underlying metabolic dysregulation, providing a mechanistic framework for further preclinical evaluation in mammalian models of epilepsy.

**Abstract:**

**Background:** Epilepsy is a chronic neurological disorder characterized by recurrent seizures, complex neurochemical, and metabolic disturbances. Parishin B, a major bioactive component of Gastrodia elata, has shown neuroprotective potential, but its systemic mechanisms remain unclear. **Methods:** A pentylenetetrazol (PTZ)-induced seizure model in zebrafish larvae was developed and used to evaluate the anti-seizure effects of Parishin B. Behavioral analysis, ELISA-based biochemical assays, integrated untargeted metabolomics with DIA-based proteomics, and qPCR were performed to decipher underlying molecular mechanisms. **Results:** Parishin B (0.0625–0.25 mg/mL) significantly alleviated PTZ-induced hyperactivity without developmental toxicity. Parishin B restored neurotransmitter balance by increasing GABA, dopamine, and norepinephrine levels while reducing 5-HT. In addition, it suppressed neuroinflammation and enhanced antioxidant capacity. Integrated multi-omics analysis revealed that Parishin B modulated key metabolic pathways, particularly the TCA cycle and lipid metabolism, and reversed the downregulation of ATP-citrate lyase (ACLY). Parishin B was also associated with the regulation of ferroptosis-related pathways, supported by changes in acsl4a and fth1a expression. qPCR results further confirmed the regulation of aclya, unc13c, and GABAergic signaling genes. **Conclusions:** Parishin B exerts anti-seizure effects through coordinated regulation of neurotransmitter homeostasis, neuroinflammation, and ACLY-associated energy–lipid metabolism, with potential involvement in ferroptosis-related processes. These findings provide molecular insights supporting Parishin B as a promising candidate for epilepsy therapy.

## 1. Introduction

Epilepsy is one of the most prevalent chronic neurological disorders, affecting approximately 70 million people worldwide [1]. It is characterized by the spontaneous recurrence of unprovoked seizures, resulting from an imbalance between excitatory and inhibitory neurotransmission [2,3]. Despite the availability of numerous anti-epileptic drugs (AEDs), approximately one-third of patients develop drug-resistant epilepsy, and many experience severe cognitive or metabolic side effects [4,5,6]. Emerging evidence suggests that the pathogenesis of epilepsy involves a complex network of neuroinflammation, oxidative stress, and systemic metabolic dysfunction [7,8]. Oxidative stress and metabolic imbalance further exacerbate seizure-related brain injury [9]. Therefore, there is an urgent need to identify novel, multi-targeted therapeutic agents with high efficacy and favorable safety profiles. Natural products, with their structural diversity and rich history of medicinal use, provide a valuable reservoir for discovering such agents [10]. Recent advances in systems biology have highlighted the critical role of metabolic reprogramming in epilepsy [11,12]. Specifically, the disruption of the Citrate cycle (TCA cycle) and subsequent lipid metabolism disorders are closely linked to neuronal hyperexcitability and ferroptosis—a form of regulated cell death driven by iron-dependent lipid peroxidation [13,14,15]. ATP-citrate lyase (ACLY), a key enzyme bridging glucose metabolism and de novo lipogenesis, has emerged as a potential metabolic hub in neurological recovery [16,17]. Whether Parishin B can alleviate seizures by targeting the ACLY-mediated energy lipid metabolism axis has not yet been explored.

Zebrafish (*Danio rerio*) larvae provide a high-throughput and physiologically relevant vertebrate model for epilepsy research due to their rapid development, transparent bodies, and high genetic homology to humans [18,19]. Pentylenetetrazol (PTZ), a competitive antagonist of the gamma-aminobutyric acid (GABA) receptor, is widely used to induce reproducible seizure-like behaviors and molecular changes in zebrafish [20,21].

*Gastrodia elata Bl.* (Tianma), a well-known traditional herbal medicine, has been used for centuries in East Asia to treat headache, dizziness, and convulsive disorders [22,23]. Parishin B, a major bioactive ester derivative of gastrodin found in G. elata, has gained increasing attention for its neuroprotective, anti-inflammatory, and antioxidant properties [23]. Previous studies have indicated that Parishin compounds can cross the blood brain barrier and modulate synaptic plasticity [24]. However, most research has focused on isolated pathways, and a systemic understanding of how Parishin B reconfigures the brain’s proteomic and metabolic landscapes to exert its anti-seizure effects remains elusive.

In the present study, we integrated behavioral assessment, biochemical analysis, and DIA-based quantitative proteomics with untargeted metabolomics to provide a comprehensive molecular insight of Parishin B’s therapeutic effects. Our results suggest that Parishin B alleviates PTZ-induced seizures by restoring neurotransmitter balance, suppressing neuroinflammation, and modulating the ACLY-associated energy–lipid metabolic axis, with potential involvement in ferroptosis-related processes. This study further complements the anti-seizure potential of Parishin B and provides systemic molecular insights into its neuroprotective mechanisms, highlighting Parishin B as a promising multi-target candidate for epilepsy therapy.

## 2. Materials and Methods

### 2.1. The Reagents and Chemicals

Parishin B (purity 98%) was purchased from Beijing Zhongnong Sichen Biotechnology Co., Ltd. (Beijing, China). Sodium valproate (VPA, purity 98%) was obtained from Shanghai Macklin Biochemical Co., Ltd. (Shanghai, China). Pentylenetetrazol (PTZ, purity 98%) was purchased from Shanghai Jizhi Biochemical Technology Co., Ltd. (Shanghai, China). ELISA kits for Glutamate (Glu), gamma-aminobutyric acid (GABA), dopamine (DA), serotonin (5-HT), norepinephrine (NE), interleukin-6 (IL-6), interleukin-1β (IL-1β), and superoxide dismutase (SOD) were purchased from Shanghai Enzyme-linked Biotechnology Co., Ltd. (Shanghai, China). The RNA extraction kit (TransZol Up Plus RNA Kit), reverse transcription kit (TransScript All-in-One First-Strand cDNA Synthesis SuperMix for qPCR), and quantitative PCR kit (PerfectStart Green qPCR SuperMix) were obtained from TransGen Biotech (Beijing, China). HPLC-grade methanol, acetonitrile, formic acid, water, and isopropanol were purchased from Fisher Scientific (Thermo Fisher Scientific, Waltham, MA, USA).

### 2.2. Zebrafish Husbandry and Breeding

Wild-type AB strain zebrafish (*Danio rerio*), aged 3–5 months, were obtained from Nanjing Yishu Pear Flower Biotechnology Co., Ltd. (Nanjing, China). Adult fish were housed and bred under standard laboratory conditions in accordance with previously established protocols [25]. Following natural spawning, fertilized embryos were collected and washed three times with sterile E3 medium (15 mmol/L NaCl, 0.67 mmol/L KCl, 0.03 mmol/L NaHCO_3_, and 0.90 mmol/L CaCl_2_; pH 7.2) to remove unfertilized eggs and residual debris. Subsequently, embryos were placed in sterile Petri dishes containing fresh E3 medium and incubated at 28 ± 1 °C under a controlled 14 h light/10 h dark photoperiod. All experimental procedures involving animals were reviewed and approved by the Animal Ethics Committee of Institute of Food Science and Technology, Chinese Academy of Agricultural Sciences (approval number: 20250225), and were conducted in strict compliance with the principles of Replacement, Reduction, and Refinement (3Rs).

### 2.3. Developmental Toxicity Assessment of Parishin B in Zebrafish Embryos

To evaluate the developmental toxicity of Parishin B, zebrafish embryos at 6 h post-fertilization (hpf) were randomly distributed into six groups and exposed to E3 medium containing Parishin B at final concentrations of 0, 0.0625, 0.125, 0.25, 0.50, and 1.0 mg/mL. The exposure medium was refreshed every 24 h, and embryos were continuously treated until 5 days post-fertilization (dpf).

Embryo survival and hatching rate were examined at 4 dpf (n = 30) under a stereomicroscope (Olympus SZX16, Olympus Corporation, Tokyo, Japan) equipped with an image analysis system. Larval body length was measured at 4 dpf (n = 10) to assess growth and developmental progression. At 5 dpf, spontaneous locomotor activity (n = 8) was recorded using an automated zebrafish behavior tracking system (Zebrabox, Noldus, China), measuring total swimming distance and average velocity over a 10 min period. Based on these assessments, non-toxic doses were selected for subsequent experiments [26,27].

### 2.4. PTZ-Induced Seizure Model and Parishin B Treatment in Zebrafish Larvae

Next, 7 dpf zebrafish larvae were individually placed into 48-well plates (one larva per well). Larvae were randomly assigned to control, PTZ model, Parishin B treatment, or VPA treatment groups (n = 8 per group). Randomisation was performed using a random number table. Larvae were pre-treated with Parishin B at 0.0625, 0.125, or 0.25 mg/mL concentrations and VPA (0.32 mg/mL; positive drug) for 60 min. Control and PTZ model groups were maintained in E3 medium.

Following pretreatment, larvae were gently rinsed with fresh E3 medium to remove residual compounds and subsequently exposed to PTZ (20 mM) to induce seizure-like hyperactivity. Locomotor behavior was continuously monitored for 40 min after PTZ exposure using the Zebrabox system. Total swimming distance and average swimming speed were quantified as indices of seizure severity [27,28]. Behavioural analysis was performed by an investigator who was blinded to the group allocation. To minimise potential confounders, the order in which different groups received treatment administration and subsequent behavioural recording was randomised across groups. The positions of the 48-well plates were also counterbalanced on the recording platform. The experiment was independently repeated three times.

### 2.5. Determination of Neurotransmitters, Inflammatory Cytokines, and Oxidative Stress Markers

For biochemical analyses, 7 dpf zebrafish larvae were randomly assigned to control, PTZ, Parishin B-treated (0.0625–0.25 mg/mL), or VPA-treated (0.32 mg/mL) groups. Larvae were pre-exposed to Parishin B, and VPA for 60 min before PTZ exposure (20 mM, 30 min). Control larvae received no treatment, whereas the PTZ group was exposed to PTZ alone.

After treatment, larvae were collected, homogenized in ice-cold phosphate-buffered saline (PBS), and centrifuged at 12,000× *g* for 15 min at 4 °C. The supernatants were collected for the quantification of glutamate (GLU), γ-aminobutyric acid (GABA), dopamine (DA), serotonin (5-HT), norepinephrine (NE), interleukin-6 (IL-6), interleukin-1β (IL-1β), tumor necrosis factor-α (TNF-α), and superoxide dismutase (SOD) using commercial ELISA kits as per manufacturers’ instructions. Samples from 30 larvae per group were pooled for each biological replicate, with six independent replicates performed (n = 6).

### 2.6. Untargeted Metabolomic Analysis

For metabolomic profiling, 7 dpf larvae were divided into three groups: control, PTZ model, and PTZ + Parishin B treatment (0.125 mg/mL). For metabolomic analysis, 90 larvae per group were pooled into six biological replicates (15 larvae per replicate). Drug exposure procedures were performed as described above for the PTZ-induced seizure model. After treatment, larvae were rapidly collected and homogenized in pre-cooled methanol/acetonitrile/water (2:2:1, *v*/*v*/*v*). Samples were centrifuged at 12,000× *g* for 15 min at 4 °C, and the supernatants were collected for analysis [29].

For LC–MS analysis, samples were analyzed using an ultra-high-performance liquid chromatography system coupled with a high-resolution mass spectrometer. Raw data files were processed using Progenesis QI software (version 4.2) for peak extraction, alignment, and normalization. Metabolite identification was achieved by matching MS/MS spectra against HMDB, METLIN, and the Majorbio metabolite database [30].

Multivariate statistical analyses, including PCA and OPLS-DA, were applied to evaluate metabolic alterations among groups. Model validity was assessed using permutation tests. Differential metabolites were screened based on VIP > 1.0 and *p* < 0.05, followed by Benjamini Hochberg false discovery rate (FDR) correction; metabolites with FDR-adjusted *p* < 0.05 were considered statistically significant. KEGG pathway enrichment analysis was performed to identify significantly affected metabolic pathways, with pathway significance determined using Benjamini Hochberg FDR correction (FDR-adjusted *p* < 0.05).

### 2.7. DIA-Based Quantitative Proteomic Analysis

Proteomic analysis was conducted using the same experimental design as mentioned in metabolomic study. For proteomics, 90 larvae per group were divided into three biological replicates (30 larvae per replicate) to ensure reproducibility. Total protein was extracted from larvae using lysis buffer, and protein concentrations were determined by BCA assay. Proteins were enzymatically digested with trypsin, desalted, and subjected to mass spectrometric analysis.

Peptide samples were analyzed on a timsTOF Pro2 mass spectrometer (Bruker, Germany) coupled to an EASY-nLC 1200 system (Thermo Fisher Scientific, Odense, Denmark) using DIA-PASEF acquisition mode. Raw data were processed using Spectronaut™ software (Version 19) against the Danio rerio UniProt database. Indexed retention time standards were used for calibration. Peptide and protein identifications were controlled at a false discovery rate (FDR) ≤ 1% at both peptide and protein levels. Protein quantification was performed using selected peptides and fragment ions under stringent quality control criteria.

Differentially expressed proteins were identified using Student’s *t*-test with a fold change threshold of >1.2, followed by Benjamini Hochberg false discovery rate (FDR) correction; proteins with FDR-adjusted *p* < 0.05 were considered statistically significant. Functional annotation and pathway enrichment analyses were conducted using Gene Ontology and KEGG databases [31].

### 2.8. Integrated Metabolomic and Proteomic Analysis

Differential metabolites (VIP > 1.0, *p* < 0.05, n = 503) and differential proteins (FC > 1.2, *p* < 0.05, n = 800) were subjected to integrated KEGG pathway enrichment analysis using the Majorbio Cloud platform with Benjamini Hochberg FDR correction. Significantly enriched pathways (FDR-adjusted *p* < 0.05) were selected as network anchors. By restricting correlation analysis to pathway-contextualized metabolite protein pairs, the multiple testing burden was reduced compared to global 503 × 800 pairwise testing. Within each pathway, Pearson correlation analysis was performed between metabolites and proteins across n = 3 biological replicates; pairs with |r| > 0.9 and FDR-adjusted *p* < 0.05 were retained and mapped onto KEGG pathway topology using the KGML module. The integrated network was visualized using the Majorbio Cloud built-in module.

### 2.9. Quantitative Real-Time PCR Analysis

Following PTZ and Parishin B treatment, total RNA was extracted from pooled larvae using a commercial RNA extraction kit. Complementary DNA was synthesized using a reverse transcription supermix. Quantitative real-time PCR was performed using SYBR Green on a real-time PCR system. For qPCR, 30 larvae per group were pooled per biological replicate, with three independent replicates performed.

Genes associated with neuroinflammation, neurotransmitter metabolism, synaptic function, and oxidative stress were analyzed, including gad2, slc32a1, gabra1, unc13c, aclya, il1b, tnfa, nfkb1, mapk3, dusp1, sod1, gpx1a, ndufs1, ucp2, th, dbh, tph1a and tph2. rpl13a was used as the internal reference gene, and relative gene expression levels were calculated using the 2^−ΔΔCt^ method. The sequences corresponding to all primer names are shown in Table 1. Primer names and sequences.

### 2.10. Statistical Analysis

The results are presented as mean ± standard error of the mean (Mean ± SEM). For statistical analysis and graph generation, GraphPad Prism 10 software was used. For behavioural and biochemical assays, one-way ANOVA followed by Benjamini Krieger Yekutieli FDR correction was used. For qPCR analysis, comparisons among multiple groups were performed using one-way ANOVA, followed by Benjamini Krieger Yekutieli false discovery rate (FDR) correction across the 18 tested genes. For metabolomic and proteomic analyses, Benjamini Hochberg FDR correction was applied due to high data dimensionality. FDR-adjusted *p* values < 0.05 were considered statistically significant. For comparisons between two groups, Student’s *t*-test was used. Data analysis was performed blind, with group codes revealed only after all statistical tests were completed.

## 3. Results

### 3.1. The Effects of Parishin B on Developmental Toxicity in Zebrafish Embryos

**Figure 1:**
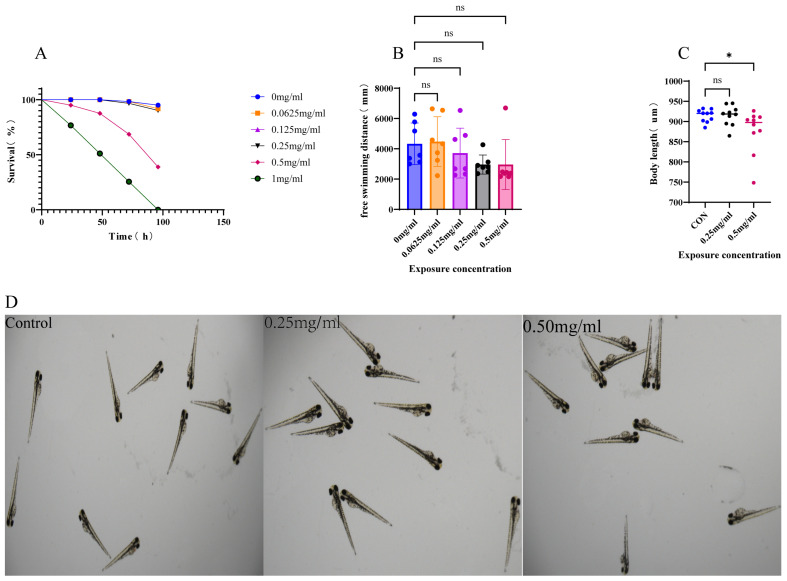
Effects of Parishin B on early development of zebrafish embryos. (**A**) Survival rate of zebrafish embryos exposed to different concentrations of Parishin B (0–1 mg/mL) from 6 hpf onward. (**B**) Spontaneous locomotor (swimming distance) activity of 5 dpf zebrafish larvae after Parishin B exposure. (**C**,**D**) Body length of zebrafish larvae measured at 4 dpf following Parishin B treatment. Data are presented as mean ± SEM. * FDR-adjusted *p* < 0.05 compared with the control group, ns, not significant.

The developmental toxicity was evaluated by measuring embryonic survival, spontaneous locomotor activity, and body length in Parishin B (0–1 mg/mL) treated larvae. As shown in Figure 1A, no significant differences in survival rate were observed in embryos treated with Parishin B at 0.0625, 0.125, or 0.25 mg/mL compared with the control group throughout the exposure period. In contrast, embryos exposed to higher concentrations of Parishin B (0.5 and 1 mg/mL) exhibited a marked decrease in survival rate (80%) at later developmental stages, indicating concentration-dependent developmental toxicity at higher doses. Spontaneous locomotor activity was assessed at 5 dpf. No significant differences in free swimming distance were observed between Parishin B-treated groups and the control group, suggesting that Parishin B does not impair basal locomotor function within the tested concentration range. Body length measurements at 4 dpf revealed that larvae treated till 0.25 mg/mL Parishin B concentrations showed no significant difference compared with controls, whereas a significant reduction in body length was observed in the 0.5 mg/mL Parishin B group (*p* < 0.05; Figure 1C,D).

Taken together, these results indicate that Parishin B exhibits no apparent developmental toxicity or behavioral impairment at concentrations ≤ 0.25 mg/mL. Therefore, Parishin B concentrations of 0.0625, 0.125, and 0.25 mg/mL were selected for subsequent pharmacological studies.

### 3.2. Parishin B Attenuates PTZ-Induced Seizure-like Hyperlocomotion in Zebrafish Larvae

**Figure 2:**
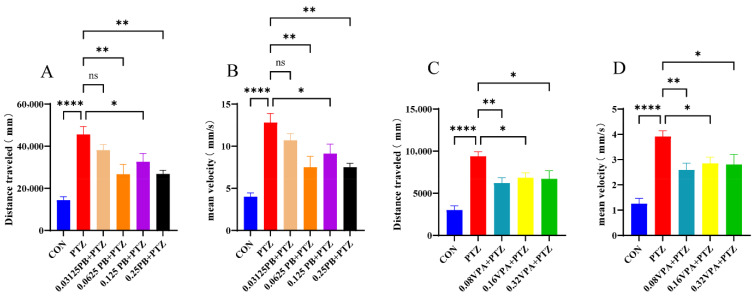
Effects of Parishin B on pentylenetetrazol (PTZ)-induced seizure-like locomotor hyperactivity in zebrafish larvae. (**A**,**B**) Total swimming distance and average swimming speed of zebrafish larvae following PTZ exposure with or without Parishin B pretreatment. (**C**,**D**) Effects of sodium valproate (VPA) on PTZ-induced changes in swimming distance and swimming speed. Data are presented as mean ± SEM. Statistical significance was determined by one-way ANOVA followed by appropriate post hoc tests. * *p* < 0.05, ** *p* < 0.01, **** *p* < 0.0001 versus PTZ group (FDR-adjusted), ns, not significant.

Seizure-like behaviors were evaluated by measuring total swimming distance and average swimming speed in zebrafish larvae following PTZ exposure. As shown in Figure 2A,B, PTZ treatment induced pronounced hyperlocomotor activity. Compared with the control group, larvae in the PTZ group exhibited significantly increased total swimming distance and average speed (*p* < 0.0001). This confirmed the successful establishment of the epileptic model.

Parishin B pretreatment ameliorated PTZ-induced behavioral abnormalities. Compared with the PTZ group, Parishin B-treated larvae showed reduced swimming distance and average speed, with statistically significant effects observed at concentrations of 0.0625–0.25 mg/mL (*p* < 0.05, *p* < 0.01), indicating effective suppression of seizure-like hyperexcitability.

As shown in Figure 2C,D, the positive control sodium valproate (VPA) also significantly reduced PTZ-induced hyperlocomotion, as evidenced by decreased swimming distance and average speed compared with the PTZ group (*p* < 0.05, *p* < 0.01).

Collectively, these behavioral results demonstrate that Parishin B, like VPA, effectively alleviates PTZ-induced seizure-like hyperactivity in zebrafish larvae.

### 3.3. Effects of Parishin B on Neurotransmitter Levels in PTZ-Treated Epileptic Zebrafish Larvae

To investigate the regulatory effects of Parishin B on neurotransmitter homeostasis, ELISA was performed to quantify levels of GLU, GABA, DA, 5-HT, and NE in 7 dpf zebrafish larvae.

**Figure 3:**
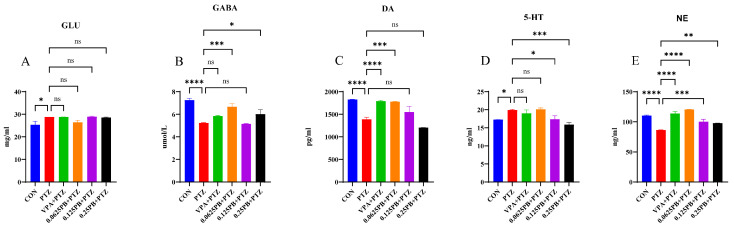
Effects of Parishin B on neurotransmitter levels in PTZ-treated zebrafish larvae. Levels of (**A**) GLU, (**B**) GABA, (**C**) DA, (**D**) 5-HT, and (**E**) NE were quantified by ELISA in 7 dpf larvae. Data are presented as mean ± SEM. * *p* < 0.05, ** *p* < 0.01, *** *p* < 0.001, **** *p* < 0.0001 compared with PTZ group (FDR-adjusted), ns, not significant.

As shown in Figure 3A–E, PTZ exposure caused marked disruption of neurotransmitter balance. Compared with the control group, PTZ treatment significantly reduced the levels of GABA, DA, and NE (Figure 3B,C,E; *p* < 0.0001), while significantly increasing the levels of 5-HT and GLU (Figure 3A,D; *p* < 0.05).

Parishin B treatment partially reversed PTZ-induced neurotransmitter abnormalities. Compared with the PTZ group, Parishin B significantly increased GABA levels (Figure 3B; *p* < 0.05, *p* < 0.001) and showed a clear restorative trend for DA levels (Figure 3C; *p* < 0.001). In addition, Parishin B significantly reduced the elevated 5-HT levels induced by PTZ (Figure 3D; *p* < 0.05, *p* < 0.001) and markedly restored NE levels (Figure 3E; *p* < 0.01, *p* < 0.001, *p* < 0.0001). However, Parishin B treatment did not significantly affect GLU levels compared with the PTZ group (Figure 3A). In the VPA-treated group, DA and NE levels were also significantly restored (Figure 3C, E; *p* < 0.0001), further validating the robustness of the model and the reliability of the assay.

These results indicate that Parishin B primarily modulates inhibitory and monoaminergic neurotransmitter systems to alleviate PTZ-induced neurotransmitter imbalance, while exerting limited effects on glutamatergic signaling.

### 3.4. Parishin B Suppresses PTZ-Induced Neuroinflammation and Oxidative Stress

**Figure 4:**
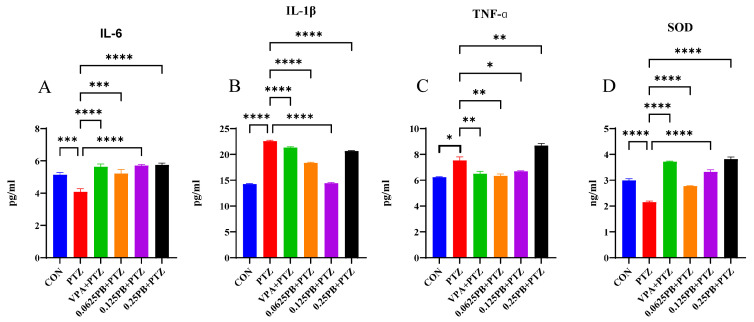
Effects of Parishin B on inflammatory cytokines and oxidative stress markers in PTZ-treated zebrafish larvae. Levels of (**A**) IL-6, (**B**) IL-1β, (**C**) TNF-α, and (**D**) SOD activity were measured by ELISA. Data are presented as mean ± SEM. * *p* < 0.05, ** *p* < 0.01, *** *p* < 0.001, **** *p* < 0.0001 versus PTZ group (FDR-adjusted).

Given the critical role of neuroinflammation in epileptogenesis, inflammatory cytokines and antioxidant enzyme activity were evaluated (Figure 4). PTZ exposure induced a pronounced inflammatory response, significantly increasing the levels of IL-1β (Figure 4B; *p* < 0.0001) and TNF-α (Figure 4C; *p* < 0.05). Parishin B pretreatment exerted dose-dependent anti-inflammatory effects. Notably, Parishin B at 0.125 mg/mL reduced IL-1β levels more effectively than the positive control VPA (*p* < 0.0001). In contrast, IL-6 levels were significantly decreased in the PTZ group (Figure 4A; *p* < 0.001), indicating impairment of endogenous neuroprotective responses. Parishin B pretreatment partially restored IL-6 levels toward normal (Figure 4A; *p* < 0.001, *p* < 0.0001). In addition, PTZ significantly reduced superoxide dismutase (SOD) activity (Figure 4D; *p* < 0.0001), indicating severe oxidative stress. Parishin B treatment significantly increased SOD activity at all tested doses (*p* < 0.0001), with antioxidant effects that were statistically superior to those of VPA.

Overall, these findings demonstrate that Parishin B confers robust neuroprotection by suppressing neuroinflammation and enhancing antioxidant defenses.

### 3.5. Parishin B Ameliorates PTZ-Induced Metabolic Disturbances in Zebrafish Larvae

**Figure 5:**
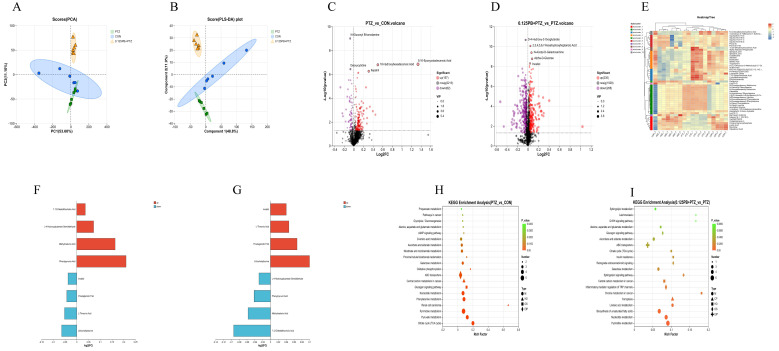
Untargeted metabolomic analysis of zebrafish larvae following PTZ and Parishin B treatment. (**A**) Principal component analysis (PCA) score plot. (**B**) Partial least squares discriminant analysis (PLS-DA) score plot. (**C**,**D**) Volcano plots showing differential metabolites in PTZ vs. CON and Parishin B + PTZ vs. PTZ comparisons. (**E**) Hierarchical clustering heatmap of significantly altered metabolites. (**F**,**G**) Differential metabolite histogram of PTZ vs. CON, differential metabolite histogram of Parishin B + PTZ vs. PTZ. (**H**,**I**) Enrichment analysis of KEGG pathway of PTZ vs. CON and KEGG pathway of Parishin B + PTZ vs. PTZ.

To further elucidate the molecular basis underlying the anticonvulsant effects of Parishin B, untargeted metabolomic analysis was performed (Figure 5). Principal component analysis (PCA; Figure 5A) and partial least squares discriminant analysis (PLS-DA; Figure 5B) revealed clear clustering and separation among the control (CON), PTZ model, and 0.125 mg/mL Parishin B-treated (Parishin B + PTZ) groups. A pronounced metabolic shift was observed between the PTZ and CON groups, whereas the Parishin B-treated group exhibited a marked tendency to cluster toward the CON group, indicating that Parishin B partially restored PTZ-induced global metabolic alterations.

Volcano plot analysis of PTZ vs. CON (Figure 5C) identified 157 significantly upregulated metabolites, including oxidative stress associated 9,10-epoxyoctadecanoic acid, and 62 significantly downregulated metabolites, such as deoxycytidine. In contrast, comparison between Parishin B + PTZ and PTZ groups (Figure 5D) demonstrated substantial metabolic normalization following Parishin B pretreatment, with 235 metabolites significantly upregulated and 268 metabolites significantly downregulated. Notably, restoration of key metabolites such as inositol and Alpha-D-glucose suggested an improvement in neuronal energy metabolism and osmotic homeostasis.

Hierarchical clustering heatmap analysis (Figure 5E) further illustrated distinct metabolic profile shifts among the groups. PTZ-treated larvae exhibited prominent upregulation of metabolites in subcluster 1, including glycerophosphocholine (Gpcho) species, phosphatidylcholine (PC) lipids, and the pro-inflammatory lipid mediator LPS (22:6), indicating PTZ-induced neuroinflammation-associated lipid accumulation. Parishin B treatment markedly reversed these changes, as reflected by a shift from high (red) to low (blue) abundance. In addition, Parishin B significantly restored energy metabolism related metabolites located in the lower region of the heatmap, such as lactic acid and choline glycerophosphate.

Bar plot analysis of representative differential metabolites (Figure 5F) revealed that PTZ treatment caused pathological elevations of 7,12-diketolithocholic acid, methylmalonic acid, phenylpyruvic acid, and L-4-hydroxyglutamate semialdehyde, reflecting excitatory neurotransmitter imbalance, organic acid dysregulation, and lipid metabolic disruption under seizure conditions. Conversely, key metabolites such as inositol, prostaglandin F3A, lithocholyltaurine and L-threonic acid were markedly reduced, suggesting that PTZ-induced epileptic seizures were accompanied by decreasing neuroregulation-related metabolites and lipid signal molecules. Parishin B pretreatment (0.125 mg/mL) effectively reversed these metabolic abnormalities (Figure 5G), providing molecular-level support for its anticonvulsant effects.

KEGG pathway enrichment analysis further demonstrated that PTZ exposure induced profound systemic metabolic dysregulation (Figure 5H). Core pathological features included significant enrichment of energy metabolism pathways, such as the citrate cycle (TCA cycle), pyruvate metabolism, and oxidative phosphorylation, indicative of seizure-induced cerebral energy imbalance. In addition, strong enrichment of pyrimidine metabolism and nucleotide metabolism suggested disruption of nucleic acid turnover. Dysregulation of phenylalanine metabolism, D-amino acid metabolism, and alanine, aspartate, and glutamate metabolism was consistent with the neurotransmitter imbalances observed in biochemical assays. Parishin B intervention (0.125 mg/mL) significantly remodeled these dysregulated pathways (Figure 5I). Parishin B markedly enriched pyrimidine metabolism, nucleotide metabolism, and the TCA cycle, indicating restoration of mitochondrial energy turnover. Furthermore, enrichment of biosynthesis of unsaturated fatty acids and sphingolipid metabolism suggested lipid profile remodeling. Notably, the appearance of ferroptosis and inflammatory mediator regulation of TRP channels pathways indicated that Parishin B modulated oxidative stress and inflammation-associated excitability at the metabolic level.

### 3.6. Parishin B Restores PTZ-Induced Proteomic Dysregulation in Zebrafish Larvae

**Figure 6:**
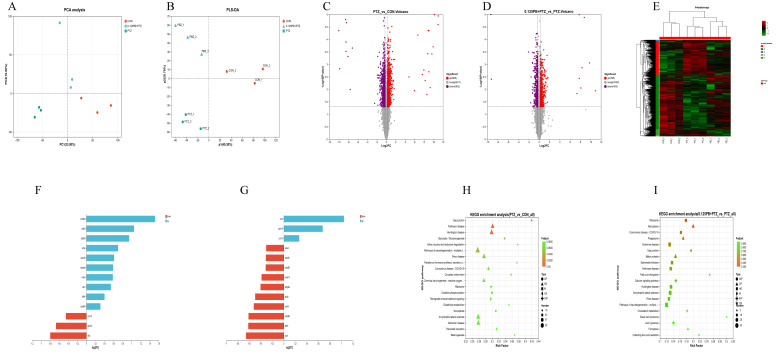
Quantitative proteomic profiling of zebrafish larvae following PTZ and Parishin B treatment. (**A**) PCA score plot. (**B**) PLS-DA score plot. (**C**,**D**) Volcano plots of differentially expressed proteins in PTZ vs. CON and Parishin B + PTZ vs. PTZ groups. (**E**) Hierarchical clustering heatmap of differentially expressed proteins. (**F**,**G**) Differential protein histogram of PTZ and CON, and differential protein histogram of Parishin B + PTZ and PTZ. (**H**,**I**) Enrichment analysis of KEGG pathway of PTZ vs. CON and KEGG pathway of Parishin B + PTZ vs. PTZ.

DIA-based quantitative proteomic analysis was conducted to identify molecular targets underlying the neuroprotective and anticonvulsant effects of Parishin B (Figure 6). PCA (Figure 6A) and PLS-DA (Figure 6B) revealed clear separation among the CON, PTZ, and Parishin B-treated groups, with Parishin B-treated samples clustering closer to the CON group, indicating partial normalization of PTZ-induced proteomic alterations.

Volcano plot analysis of PTZ vs. CON identified 666 significantly upregulated and 982 significantly downregulated proteins (Figure 6C), demonstrating severe disruption of proteomic homeostasis following seizure induction. Parishin B treatment (Parishin B + PTZ vs. PTZ) significantly regulated a total of 800 differentially expressed proteins, including 395 upregulated and 405 downregulated proteins (Figure 6D). Hierarchical clustering heatmap analysis (Figure 6E) further confirmed that Parishin B treatment effectively reversed PTZ-induced global proteomic changes.

As shown in Figure 6F, PTZ exposure significantly altered the expression of proteins related to neuronal structure and synaptic function. Several cytoskeleton-associated proteins, including tubb4bl, tubb5, tubb2b, tuba2, tuba8l2, and MAP, as well as ucp1, arhgdia, and casq1b, were upregulated, whereas the synaptic vesicle associated protein unc-13 and the signaling proteins nlk2 and ptpn12 were markedly downregulated, indicating pronounced cytoskeletal remodeling and synaptic dysfunction under epileptic conditions. Parishin B treatment (0.125 mg/mL) significantly attenuated these PTZ-induced alterations (Figure 6G), reversing the downregulation of aclyb, arhgdia, nlk2, and unc-13, and suppressing the abnormal upregulation of tubb family proteins and ucp1, indicating that Parishin B has a strong recovery effect on PTZ-induced protein disorder, especially by stabilizing the cytoskeleton and maintaining synaptic protein homeostasis.

Pathway enrichment analysis revealed that PTZ-induced proteomic alterations were primarily associated with neuronal electrical communication and signal transmission disorders (Figure 6H). The most significantly enriched pathway with the highest enrichment factor was gap junction, indicating disruption of electrical synaptic transmission. In addition, enrichment of Parkinson disease, Huntington disease, and glycolysis/gluconeogenesis pathways suggested seizure-associated neurodegenerative risk and metabolic collapse. Extensive enrichment of the ribosome pathway reflected severe impairment of the protein translation machinery. In contrast, Parishin B treatment activated multiple restorative pathways (Figure 6I). Prominent enrichment of the ribosome pathway indicated robust recovery of protein synthesis capacity. Significant modulation of necroptosis suggested protection against parenchymal injury. Enrichment of fatty acid elongation and ferroptosis pathways aligned with enhanced antioxidant defenses. Restoration of gap junction and axon guidance pathways, together with normalization of unc-13 expression, indicated improved synaptic structural integrity and neuronal signaling.

### 3.7. Integrated Metabolomic and Proteomic Analysis of Parishin B-Treated PTZ-Induced Zebrafish Larvae

**Figure 7:**
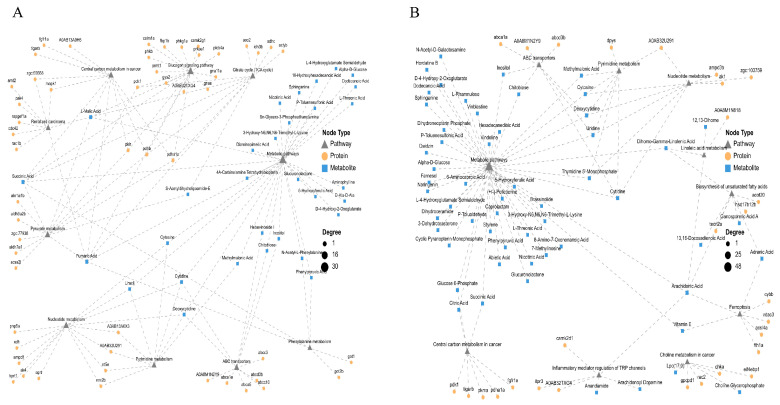
Integrated network analysis of metabolomic and proteomic data. (**A**) Network illustrating PTZ-induced dysregulated pathways. (**B**) Network showing Parishin B-mediated restoration of metabolic and signaling pathways. Gray triangles represent pathways, yellow circles represent proteins, and blue squares represent metabolites.

The PTZ-induced dysregulated network (Figure 7A) exhibited a highly interconnected architecture centered on energy metabolism, with metabolic pathways serving as major network hubs. Energy metabolism related proteins (aclyb) formed dense interaction networks with multiple key intermediates of the TCA cycle, including succinic acid and fumaric acid, suggesting that epileptic seizures are accompanied by pronounced systemic disturbances and depletion of energy metabolism. Meanwhile, metabolites associated with nucleotide metabolism showed extensive clustering, reflecting increased nucleic acid metabolic demand under seizure-related stress.

In contrast, the repair network formed following Parishin B treatment (Figure 7B) displayed prominent features of defensive metabolic reprogramming. Parishin B reshaped the network connectivity of energy metabolism related nodes, including citric acid and succinic acid, through modulation of key central carbon metabolism proteins such as pdha1a and pkma. Along the lipid metabolic axis, modules related to unsaturated fatty acid biosynthesis and linoleic acid metabolism were coupled via the shared metabolite arachidonic acid and exhibited tight functional association with the ferroptosis pathway. Ferroptosis-related proteins (acsl4a, fth1a, and vdac3), together with the antioxidant node vitamin E, collectively constituted a coordinated regulatory network against lipid peroxidation. In addition, enrichment of the inflammatory mediator regulation of TRP channels pathway suggests that Parishin B may further modulate seizure-related neuronal excitability by regulating inflammation-associated metabolites such as anandamide.

### 3.8. qPCR Validation of Key Differentially Expressed Genes

**Figure 8:**
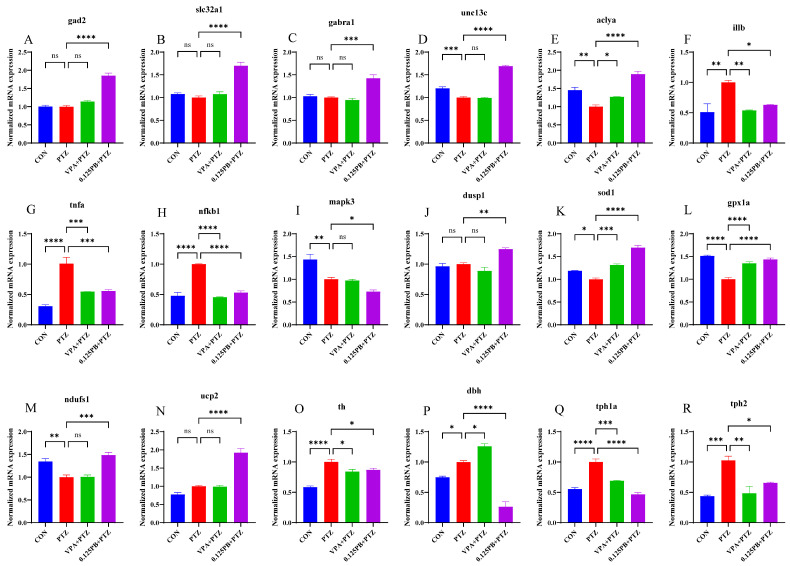
Quantitative real-time PCR validation of key differentially expressed genes. (**A**–**E**) Genes related to inhibitory neurotransmission and synaptic function. (**F**–**J**) Genes associated with inflammatory signaling. (**K**–**N**) Genes involved in oxidative stress response and mitochondrial function. (**O**–**R**) Genes related to monoaminergic neurotransmitter metabolism. Data are presented as mean ± SEM. * *p* < 0.05, ** *p* < 0.01, *** *p* < 0.001, **** *p* < 0.0001 compared with PTZ group (FDR-adjusted), ns, not significant.

To validate the reliability of the proteomic and metabolomic findings and further elucidate the molecular mechanisms underlying Parishin B-mediated seizure attenuation, quantitative PCR was performed to assess the expression of key genes involved in neurotransmission, inflammation, oxidative stress, and energy metabolism (Figure 8). Overall, mRNA expression patterns were highly consistent with multi-omics results.

Regarding inhibitory neurotransmission and synaptic homeostasis (Figure 8A–E), PTZ treatment induced either unchanged or downward trends in the expression of gad2, slc32a1, and gabra1. In contrast, synaptic priming gene unc13c and the metabolic synaptic regulatory gene aclya were significantly downregulated in the PTZ group (*p* < 0.01, *p* < 0.001). Parishin B treatment significantly upregulated all these genes (*p* < 0.001, *p* < 0.0001), with particularly strong reversal of aclya and unc13c suppression.

In terms of inflammatory and stress responses (Figure 8F–J), PTZ exposure markedly increased the mRNA levels of pro-inflammatory genes il1b, tnfa, and the transcription factor nfkb1 (*p* < 0.01, *p* < 0.0001). Parishin B treatment significantly suppressed these elevations (*p* < 0.05, *p* < 0.001, *p* < 0.0001). Additionally, Parishin B downregulated the stress-related kinase mapk3 while restoring expression of the dual-specificity phosphatase dusp1 (*p* < 0.05, *p* < 0.01), indicating precise modulation of MAPK signaling.

With respect to oxidative stress and mitochondrial protection (Figure 8K–N), PTZ significantly reduced the expression of sod1, gpx1a, and ndufs1 (*p* < 0.05, *p* < 0.01, *p* < 0.0001). Parishin B intervention markedly increased the mRNA levels of sod1, gpx1a, and mitochondrial function related genes ndufs1 and ucp2 (*p* < 0.001, *p* < 0.0001), consistent with enrichment of the peroxisome pathway in proteomic analysis.

Finally, genes involved in monoaminergic neurotransmitter metabolism (Figure 8O–R), including th, dbh, tph1a, and tph2, were significantly upregulated in the PTZ group (*p* < 0.05, *p* < 0.001, *p* < 0.0001), reflecting monoaminergic dysregulation during seizures. Parishin B treatment significantly suppressed the abnormal overexpression of these genes (*p* < 0.05, *p* < 0.001, *p* < 0.0001), with particularly strong effects on dbh and tph1a.

Collectively, qPCR validation corroborated the robustness of the multi-omics datasets and demonstrated that Parishin B exerts potent neuroprotective and anticonvulsant effects through coordinated regulation of energy metabolism centered on aclya, enhancement of GABAergic inhibitory signaling, and suppression of NF-κB mediated inflammatory cascades.

## 4. Discussion

The pathophysiology of epilepsy is characterized by a complex interplay of neuronal hyperexcitability, metabolic exhaustion, and a persistent neuroinflammatory state [7,32,33]. In this study, we observed that Parishin B was associated with attenuation of PTZ-induced seizures in zebrafish larvae. Our data suggest that Parishin B was associated with multi-target regulatory network effects, including alterations in neurotransmitter balance, metabolic reprogramming, particularly involving the ACLY-related energy–lipid axis, and potential modulation of ferroptosis. This study, for the first time, applied a multi-omics approach to generate hypotheses regarding the molecular mechanisms by which Parishin B may correlate with seizure alleviation in zebrafish larvae. Moreover, this study further identifies Parishin B as a potential candidate to be further investigated as an anti-epileptic agent.

The observed correlation between Parishin B treatment and a shift towards excitatory inhibitory (E/I) balance suggests a potential mechanism underlying the effects of Parishin B. Seizure activity is typically initiated by a collapse of GABAergic inhibition [34], a deficit that was positively correlated with Parishin B treatment, which was further associated with increased GABA levels and upregulation of critical synthesis and transport genes such as gad2 and slc32a1 [35,36]. Furthermore, Parishin B was associated with normalized expression of synaptic genes, including unc13c, suggesting a potential supportive role in synaptic function. As a critical member of the Munc13 priming complex, UNC-13 is essential for synaptic vesicle priming [37]. Our findings indicate that PTZ-induced stress may impair vesicle priming, and this impairment correlated with Parishin B treatment. The observed correlation between Parishin B levels and the expression of unc13c and other synaptic and gap junction proteins raises the possibility that it may be involved in the stabilization of inhibitory synaptic transmission. This effect is complemented by correlations with the monoamine system, where Parishin B treatment was associated with restored dopamine and norepinephrine levels, suggesting a possible systemic dampening of neuronal hypersynchronization [38,39].

Concurrently, the associations of Parishin B treatment with reduced inflammatory and oxidative stress markers suggest a potential to mitigate secondary neuronal injury. The transition from acute seizures to chronic epileptic states is often mediated by neuroinflammatory cascades [40,41]. Our qPCR data showed that Parishin B was associated with reduced expression of il1b, tnfa, and nfkb1 with upregulation of dusp1 [42,43]. The correlation between Parishin B and partial restoration of IL-6 levels could indicate a neurotrophic effect [44]. When considered together, the correlations among Parishin B treatment, enhanced SOD activity, and upregulation of sod1 and gpx1a are consistent with a model in which oxidative stress is alleviated [45].

A central finding of our integrated multi-omics analysis is the identification of ATP-citrate lyase (ACLY) as a potential metabolic node that may link energy imbalance to biosynthetic processes. Seizures cause energy burden, which leads to metabolic stress characterized by TCA cycle disorder [46]. ACLY is the rate-limiting enzyme that generates cytosolic acetyl-CoA from mitochondrial citrate [47]. Parishin B treatment was significantly correlated with the restoration of its downregulation, which may facilitate a shift toward the biosynthesis of unsaturated fatty acids [17]. This ACLY-driven generation of acetyl-CoA is essential not only for de novo lipogenesis to repair damaged neuronal membranes but also aligns with the observed enrichment of related lipid metabolic pathways [48]. The association between Parishin B treatment and changes in the aclya-pdha1a-pkma axis is consistent with the hypothesis that it could influence the availability of precursors for lipid repair, though direct causal evidence is lacking.

This metabolic reconstruction is intrinsically linked to the potential inhibition of ferroptosis, a form of regulated cell death driven by iron-dependent lipid peroxidation [49]. Our integrated metabolomic and proteomic analysis revealed that the expression of key players in this pathway correlated with Parishin B treatment, including ACSL4a, the iron-storage protein FTH1a [50,51], and showed associations with antioxidant nodes like Vitamin E into the regulatory network [52]. Thus, it is plausible that Parishin B is associated with the counteraction of lipid peroxidation processes. This is corroborated by the reduction in pro-inflammatory lipids and the observed correlation with restoration of Inositol—a metabolite that supports osmotic balance and membrane stability [53,54]. Consequently, the observed link between Parishin B treatment and modulation of ferroptosis-related markers suggests that inhibiting this process may represent an important protective mechanism against neuronal loss.

Furthermore, the correlational findings regarding Parishin B extend to the restoration of nucleotide metabolism and ribosomal function. The disturbance of nucleic acid metabolism observed in the seizure model reflects widespread cellular stress [55]. Parishin B’s intervention in pyrimidine and nucleotide pathways, is consistent with a model in which Parishin B is associated with the reactivation of the neuronal protein synthesis machinery [56]. Additionally, the indicated stabilization of the endocannabinoid system, specifically the recovery of anandamide-associated pathways, provides a further layer of synaptic modulation, potentially dampening excessive calcium influx [57,58].

From a phytochemical perspective, Parishin B may represent a natural “prodrug” strategy [59,60]. Its complex ester structure could theoretically confer advantages in stability and delivery [61]. Upon metabolic cleavage, its components may act synergistically: the gastrodin moiety may contribute to neurotransmitter system stabilization, while the citrate moiety could potentially fuel the ACLY-mediated biosynthetic axis [14]. This inherent dual-action profile is consistent with the observed correlations between Parishin B treatment and alterations in pathways related to energy depletion, lipid membrane damage, and neuroinflammation. The consistency between our proteomic, metabolomic, and transcriptomic findings supports the robustness of this multi-layered regulatory network.

Despite these insights, several limitations of the current study should be considered. In line with the correlational nature of our multi-omics findings, although our results demonstrate robust anti-seizure effects of Parishin B in zebrafish larvae, the study relied solely on acute PTZ-induced seizures in 7 dpf zebrafish, which may not fully replicate the complexity of chronic or mammalian epilepsy. Moreover, Parishin B was tested at limited concentrations and exposure durations, leaving long-term safety and chronic efficacy unclear. While behavioral (n = 8), biochemical (n = 6), and omics (n = 3–6) sample sizes were modest, stringent FDR correction (Benjamini Hochberg for multi-omics; Benjamini Krieger Yekutieli for qPCR and other assays) was applied to control false discovery rates; nevertheless, larger cohorts would enhance statistical power to detect subtle effects. Additionally, while ACLY, unc13c, and ferroptosis pathways were identified as key nodes, direct functional validation was not performed, so their causal roles remain to be confirmed. Finally, the translational relevance of Parishin B is still speculative, as its metabolism, blood brain barrier penetration, and pharmacokinetic properties in mammals are unknown.

In conclusion, our multi-omics study provides a hypothesis-generating molecular map correlated with Parishin B’s anti-seizure association, indicating that its effects may be linked to the ACLY-mediated energy lipid axis. The co-variation of behavioral recovery, neurotransmitter balance, and the mitigation of neuroinflammation and ferroptosis with Parishin B treatment suggests an integrated effect that could potentially target both acute symptoms and underlying metabolic dysregulation associated with epilepsy. These results provide preliminary evidence that identifies Parishin B as a multi-target candidate for epilepsy and related neurological disorders.

## 5. Conclusions

Treatment with Parishin B was significantly associated with alleviation of PTZ-induced seizure-like behaviors in zebrafish larvae and was associated with restoration of neurotransmitter balance, attenuation of neuroinflammation, and improvement of oxidative stress. Integrated metabolomic and proteomic analyses suggest that Parishin B treatment correlates with changes in energy and lipid metabolism, with converging evidence suggesting that the ACLY-mediated energy–lipid metabolic axis may represent an important regulatory hub linked to its neuroprotective associations. In addition, Parishin B treatment is associated with alterations in ferroptosis-related pathways, suggesting a potential association with reduced lipid peroxidation linked neuronal damage. Overall, these findings provide correlative molecular evidence that generate the hypothesis of Parishin B as a promising multi-target candidate for epilepsy therapy and suggest the therapeutic relevance of targeting metabolic–synaptic coupling in seizure disorders.

## Figures and Tables

**Table 1:** Primer names and sequences.

Gene Name	Primer Sequence
rpl13a	F: TCTGGAGGACTGTAAGAGGTATGC R: AGACGCACAATCTTGAGAGCAG
gad2	F: CAAGAAGCATGACGTCTGGA R: CTGCATCAGTCCCTCCTCTC
slc32a1	F: GCCACTACCGTCACCAATAAGT R: CCTGTGGTCATAGTCCAGATCA
gabra1	F: GTAGCCTATGCCACAGCCAT R: TCTTTTGCTTTTCTGGCACCAC
unc13c	F: ATCGACCTCTCCAAGTACCG R: CGGTTGATTTTAAGTCATGAAGTCT
aclya	F: AGACCTGATCTCCAGCCTCACATC R: ATGCCACTGTCGAATGCCTTACTG
il1b	F: GAACAGAATGAAGCACATCAAACC R: ACGGCACTGAATCCACCAC
tnfa	F: AGACCTTAGACTGGAGAGATGAC R: CAAAGACACCTGGCTGTAGAC
nfkb1	F: AGGCCAAAGACACTGTTCGG R: GGAAAGGTTGTGGGGTCCAT
mapk3	F: ATGGCGGAATCGGGCAGTAGCGCGGC R: ATTATCAAAAGCTGAGCAGACCATC
dusp1	F: CTCTGTATGATCAGGGTGGCCC R: CGGTGATCCCCAACATGTCC
sod1	F: AAGAAGCCAGTGAAGGTGACT R: CGTGTCTCACACTATCGGTTG
gpx1a	F: AGATGTCATTCCTGCACACG R: AAGGAGAAGCTTCCTCAGCC
ndufs1	F: GCGAGCAATATGGTTGAAG R: AGACACATACGGCAGTTAC
ucp2	F: CACTGGACACCGCAAAAGTT R: CGTACCAAAGACCCCTCGAT
th	F: GCTCTCAGCACGCGATTTTT R: TCATGGACGCAATCCGGTTC
dbh	F: TGCCTATGGGAGGAGAAGGA R: TAAACGAATGCCCGAGGAGT
tph1a	F: CAGTTCAGTCAGGAGATTGG R: GACAGTGCGTGCTTCAG
tph2	F: TCCTCCATGGACATCCTTTAATCG R: GCTTCTCCTGAAAGAGCCTCA

## Data Availability

The original contributions presented in this study are included in the article. Further inquiries can be directed to the corresponding author.
